# Simultaneous or staged operation for tandem spinal stenosis: surgical strategy and efficacy comparison

**DOI:** 10.1186/s13018-021-02357-x

**Published:** 2021-03-24

**Authors:** Junming Cao, Xianda Gao, Yipeng Yang, Tao Lei, Yong Shen, Linfeng Wang, Zheng Tian

**Affiliations:** Department of Orthopedics, The Third Hospital of Hebei Medical University, The Key Laboratory of Orthopedic Biomechanics of Hebei Province, 139 Ziqiang Road, Shijiazhuang, Hebei 050051 P. R. China

**Keywords:** Tandem spinal stenosis, Cervical, Lumbar, Simultaneous decompression, Staged surgery

## Abstract

**Background:**

Tandem spinal stenosis (TSS) has a complex clinical presentation, and there is no consensus on the optimal surgical strategy. This study retrospectively compared the efficacy of different staged operations and simultaneous decompression for patients with TSS.

**Methods:**

We reviewed data from 132 patients with TSS who received surgical procedures from January 2011 to June 2018. Patients were classified into three groups according to the most symptomatic area of compression (group C: first-stage surgery for cervical compression; group L: first-stage surgery for lumbar compression; group CL: simultaneous surgery for both). Medical records were reviewed for age, gender, comorbidities, operation time, combined estimated blood loss, and time of hospitalization. The JOA-C, JOA-L, NDI, and ODI scores, and complications were also examined.

**Results:**

Postoperative outcomes were followed for 32.1 ± 5.4 months. There were significant differences in the re-operation rate and the interval time between the two types of staged operations (*p* = 0.005 and *p* = 0.001, respectively). There were no significant differences in gender (*p* = 0.639), operation time (*p* = 0.138), combined estimated blood loss (*p* = 0.116), or complications (*p* = 0.652) among the three groups, while the simultaneous group was significantly younger (*p* = 0.027), with fewer comorbidities (*p* < 0.001) and a shorter hospitalization time (*p* < 0.001). At the final follow-up, the JOA-C and JOA-L scores were increased, while the NDI and ODI scores were decreased, compared with the preoperative scores.

**Conclusions:**

TSS can be effectively managed by either simultaneous or staged decompressions. First-stage surgery for cervical stenosis significantly lowers the requirement for second-stage lumbar surgery. One-stage simultaneous decompression is safe and effective with the advantage of reduce hospitalization time, without an increase in operative time or bleeding. However, the surgical indications should be strictly controlled and is recommended for younger patients with fewer comorbidities.

## Introduction

Tandem spinal stenosis (TSS) refers to narrowing of the spinal canal diameter in at least two distinct regions of the spine, most commonly the lumbar and cervical regions. TSS patients classically manifest with a constellation of cervical spondylosis and concomitant lower-extremity symptoms secondary to lumbar stenosis. In 1964, Teng et al. [1] first described combined cervical and lumbar spondylosis in 12 patients, while Dagi et al. [2] coined the term TSS as a clinical triad of symptoms of intermittent lower-extremity claudication, gait disturbance, and upper and lower motor neuron signs. The prevalence of TSS ranges from 0.12 to 34% [3–7].

As the symptoms of TSS may present in both the upper and lower extremities, the optimal surgical strategies remain controversial. This includes how to identify the most symptomatic stenosis, which procedure (two-staged or single-stage surgery) is more effective, and which stenosis should be treated first in the staged operation. In this retrospective study, we reviewed 132 patients diagnosed with TSS between January 2011 and June 2018 in the Third Hospital of Hebei Medical University. The aim of this study was to compare outcomes after simultaneous decompression of the cervical and lumbar spine versus different staged operations and to summarize a clearly defined surgical strategy.

## Methods

After Institutional Review Board approval and in accordance with the STROBE statement, the records of patients with symptomatic TSS who underwent spinal decompression surgery from January 2011 to June 2018 were retrospectively reviewed for demographics including age, gender, and comorbidities. These patients were initially admitted with symptomatic cervical spondylosis or degenerative lumbar disease, but abnormal signs and additional nerve compression in the lumbar or cervical segments were found during the physical examination and further imaging examinations. Diagnosis of lumbar spinal stenosis or cervical spondylosis was then performed. Magnetic resonance images were obtained in all patients. We adopted the stenosis grading systems proposed by Kang et al. [8] and Lee et al. [9] using cervical T2-weighted sagittal images and lumbar T2-weighted axial images, respectively. Both grading systems have moderate-to-excellent reliability and are widely used in clinical studies [10, 11].

The inclusion criteria were as follows: (1) the main symptoms included cervical myelopathy or intractable radiculopathy, such as numbness or pain in the upper extremities, hand clumsiness, or gait disturbances, coupled with neurogenic claudication, low back pain, or lumbar radiculopathy; (2) constellations of positive upper and lower motor neuron signs, including decreased force of the upper limbs, Hoffman sign, or unsteady gait, and with abnormal sensory or muscular strength in the area of lumbar innervation, but without positive clonus or hyperreflexia [2, 3, 6, 12, 13]; (3) all patients exhibited grade 1–3 cervical canal stenosis [8] and grade 1–3 lumbar central canal stenosis [9]; and (4) for patients with complex symptoms (*n* = 36), electrophysiological techniques (motor- and sensory-evoked potentials and electroneuromyography) were performed to determine the region most affected. Patients with spinal tumors, infection, fracture, congenital deformations, amyotrophic lateral sclerosis, or demyelination disorders were excluded. A total of 150 patients undergoing a simultaneous or staged operation for concurrent cervical and lumbar degenerative disease were included in this study. After excluding 18 patients with incomplete records or who were unable to be followed up, the remaining 132 patients had a mean follow-up period of 32.14 ± 5.4 months, with 81 men and 51 women (38%). The average patient age was 62.7 ± 7.6 (46–79) years. Eighty-one patients had at least one coexisting disease that did not affect the surgical and anesthetic indications, which included diabetes mellitus (*n* = 6), hypertension (*n* = 23), ischemic heart disease (*n* = 19), lacunar infarction (*n* = 8), rheumatoid arthritis (*n* = 3), hypothyroid (*n* = 7), and obesity (*n* = 15).

**Fig. 1:**
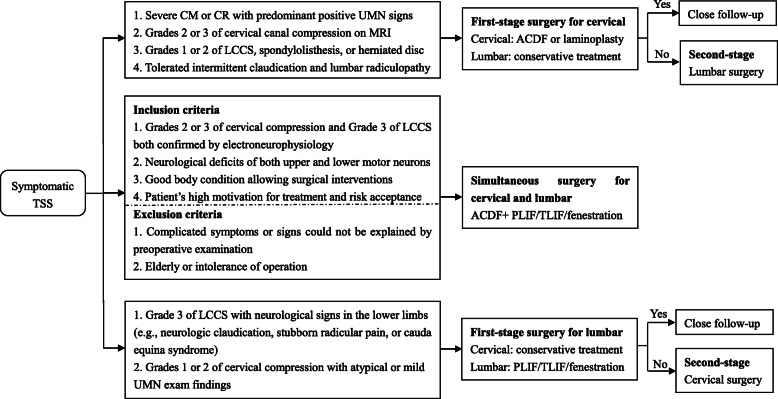
Surgical algorithm for the management of tandem spinal stenosis. Note: ACDF, anterior cervical discectomy and fusion; CM, cervical myelopathy; CR, cervical radiculopathy; LCCS, lumbar central canal stenosis; PLIF, posterior lumber interbody fusion; TLIF, transforaminal lumber interbody fusion; UMN, upper motor neuron

The responsible spinal segments were clinico-radiologically diagnosed. We used a comprehensive algorithm to address TSS in a staged or simultaneous fashion (Fig. 1). Generally, cervical decompression was performed first (group C) for patients with severe cervical myelopathy or radiculopathy but mild lumbar degenerative disease. Lumbar surgery was performed first (group L) in the presence of severe lumbar central canal stenosis, lumbar radiculopathy, and cauda equina syndrome but with atypical or mild cervical spondylosis. After the first-stage decompression for the most symptomatic lesions, patients were cautiously watched for symptoms from the non-operated region. If such symptoms were debilitating, second-stage surgery was performed. Nevertheless, the criteria for one-stage combined cervical and lumbar decompression were strictly controlled (Fig. 1).

All surgeries were performed by the same orthopedic team. Surgical options for cervical spondylosis were anterior cervical discectomy and fusion (ACDF) or laminoplasty. ACDF was performed for patients with a pathological extent that did not exceed three intervertebral levels and without ossification of the posterior longitudinal ligament (OPLL). Laminoplasty was performed for patients with a pathological extent greater than three levels or with a long-segment OPLL. Depending on the pathological location and segmental instability, surgical procedures for lumbar spondylosis were posterior lumber interbody fusion (PLIF) for patients with central stenosis, spondylolisthesis, or bilateral symptoms, transforaminal lumber interbody fusion (TLIF) for patients with unilateral narrowing of the foramen or lateral recess, and fenestration for patients with unilateral lumbar disc herniation without segment instability. In simultaneous operations, ACDF was performed first supinely, followed by lumbar prone decompression with protection using a soft neck collar.

Medical records were reviewed to evaluate the operation time (ORT), combined estimated blood loss (EBL), time of hospitalization, and complications. ORT, EBL, and time of hospitalization represent the combined total for both the cervical and lumbar cases, whether simultaneous or staged. Clinical symptoms were evaluated using the Japanese Orthopedic Association (JOA) score for cervical myelopathy (JOA-C; total of 17 points), JOA score for lumbar pain (JOA-L; total of 29 points), and the Neck Disability Index (NDI; total of 50 points) in the cervical spine and the Oswestry Disability Index (ODI; total of 20 points) for back pain-related disability. Surgical improvement was measured by assessing the differences between preoperative and final follow-up.

### Statistical analysis

Statistical analysis was performed using SPSS version 16.0 software (SPSS Inc., Chicago, Illinois, USA). All data are presented as the mean ± standard deviation. Comparisons of continuous variables were performed using the Student’s *t* test or the Mann–Whitney *U* test, as appropriate. Comparisons of categorical variables were performed using the chi-square test or the Fisher’s exact test, as appropriate. Comparisons of multiple group variables were performed using analysis of variance or the Mann–Whitney *U* test, while multiple comparisons were performed using the Bonferroni test. A *p* value < 0.05 was considered statistically significant.

## Results

### Cervical surgery was performed first (group C)

Of the 89 patients who received cervical surgery first, there were 56 (62.92%) patients with cervical myelopathy, 5 (5.62%) with radiculopathy, and 28 (31.46%) with myeloradiculopathy. Grade 3 cervical canal compression was noted in 67 patients (75.28%) and grade 2 in 22 patients (24.72%). Eighty-one patients received ACDF and eight received laminoplasty. After the cervical operation, these patients were treated conservatively using bed rest, lumbar bracing, physical therapy, and non-steroidal anti-inflammatory drugs to relieve the lumbar symptoms. Thirty-four patients (38.20%; group C1) had disappearance or alleviation of lower back pain or leg radicular pain or numbness and did not require a second-stage surgery. However, 55 patients (61.80%; group C2) had exacerbation of previous symptoms of lumbar stenosis, such as progressive neurologic claudication, weakness in a single or multiple lumbar nerve root distribution, or cauda equina syndrome. Thus, second-stage lumbar surgery was performed after an interval of 5.58 ± 1.93 months. Seventeen patients received TLIF, 31 received PLIF, and seven received fenestration. The follow-up is summarized in Table 1.

**Table 1:** Scores in patients underwent first-stage cervical decompression (group C)

	Group C1	Group C2
Number	34	55
Age (years)	62.0 ± 8.4	63.3 ± 7.1
Gender		
Male	22	32
Female	12	23
No. of coexisting disease (%)	20 (58.8%)	40 (72.7%)
Cervical JOA score		
Preoperation	8.62 ± 1.63	8.55 ± 1.84
Follow-up	12.94 ± 1.86^1^	12.82 ± 2.05^1^
NDI		
Preoperation	19.27 ± 7.62	21.44 ± 8.58
Follow-up	8.41 ± 4.19^1^	10.24 ± 4.35^1^
Lumber JOA score		
Preoperation	17.15 ± 2.00	15.35 ± 2.16^2^
Follow-up	19.26 ± 2.02^1^	24.51 ± 1.93^1,2^
ODI		
Preoperation	28.09 ± 8.92	30.27 ± 4.93
Follow-up	21.85 ± 8.31^1^	14.36 ± 5.93^1,2^

Note: ^1^Compared with preoperation, *P* < 0.05; ^2^compared with group C1, *P* < 0.05

There were no significant differences in age, gender, rate of coexisting disease, or preoperative JOA-C, NDI, and ODI between group C1 and group C2. However, the preoperative JOA-L of group C1 was significantly higher than that of group C2 (*p* < 0.001). In group C1, the JOA-C was significantly increased (*p* < 0.001) and the NDI significantly decreased (*p* < 0.001) compared with preoperatively, while both the JOA-L (*p* < 0.001) and ODI (*p* < 0.001) were improved after conservative treatment. In group C2, all indexes showed significant improvement at final follow-up (*p* < 0.001). At final follow-up, there were no significant differences in JOA-C (*p* = 0.776) or NDI (*p* = 0.055) between group C1 and group C2, while there were significant differences in the JOA-L (*p* < 0.001) and ODI (*p* < 0.001).

### Lumbar surgery was performed first (group L)

Of the 29 patients who received lumbar surgery first, there were 12 (41.38%) patients with intermittent claudication, two (6.90%) with lumbar radiculopathy, and 15 (51.72%) with both. Four patients received TLIF and 25 patients received PLIF. After the lumbar operation, these patients were treated conservatively by bracing the neck, physical therapy, and neurotrophic drugs including mecobalamine and mouse nerve growth factor to relieve cervical symptoms. Only three patients (10.34%; group L1) had resolution of neck pain or upper-extremity radicular pain or tingling and did not require a second-stage surgery. The second cervical surgery was performed in 26 patients (89.66%; group L2) after an interval of 3.96 ± 1.89 months for exacerbation of previous myelopathic symptoms, including hand clumsiness, gait disturbance, coordination difficulty, and sensory deficits. Twenty-four patients received ACDF and two patients received laminoplasty. The follow-up is summarized in Table 2.

**Table 2:** Scores in patients underwent first-stage lumbar decompression (group L)

	Group L1	Group L2
Number	3	26
Age (years)	62.7 ± 3.5	64.6 ± 7.5
Gender		
Male	2	15
Female	1	11
No. of coexisting disease (%)	2(66.7%)	17(65.4%)
Lumber JOA		
Preoperation	11.67±2.08	11.77±2.16
Follow-up	21.67±3.06^1^	19.77±2.23^1^
ODI		
Preoperation	29.33±4.51	25.27±9.38
Follow-up	16.33 ± 3.51^1^	13.73 ± 5.83^1^
Cervical JOA		
Preoperation	13.00 ± 1.00	10.08 ± 2.10^2^
Follow-up	13.67 ± 1.15	13.50 ± 1.94^1^
NDI		
Preoperation	14.33 ± 2.52	15.50 ± 7.27
Follow-up	12.33 ± 2.18	8.88 ± 3.54^1^

Note: ^1^Compared with preoperation, *P* < 0.05; ^2^compared with group L1, *P* < 0.05

There were no significant differences in age, gender, rate of coexisting disease, and preoperative JOA-L, NDI, or ODI between group L1 and group L2. However, the preoperative JOA-C of group L1 was significantly higher than that for group L2 (*p* = 0.026). In group L2, the JOA-L was significantly increased (*p* < 0.001) and ODI was significantly decreased (*p* < 0.001) compared with preoperatively. However, there was no improvement in the JOA-C (*p* = 0.184) or the NDI (*p* = 0.074) after conservative treatment. In group L2, all indices showed significant improvement at final follow-up (*p* < 0.001). At final follow-up, there were no differences in JOA-L (*p* = 0.188), ODI (*p* = 0.460), JOA-C (*p* = 0.887), or NDI (*p* = 0.113) between group L1 and group L2.

When comparing the two types of staged surgeries (group C2, group L2), the requirement for a second-stage surgery in group C (55/89 [61.80%], group C2) was significantly lower than that in group L (26/29 [89.66%], group L2; *p* = 0.005). The average interval between surgeries was significantly lower in group L2 (3.96 ± 1.89 months) compared with group C2 (5.58 ± 1.93 months; *p* = 0.001).

### Simultaneous surgery was performed (group CL)

**Fig. 2:**
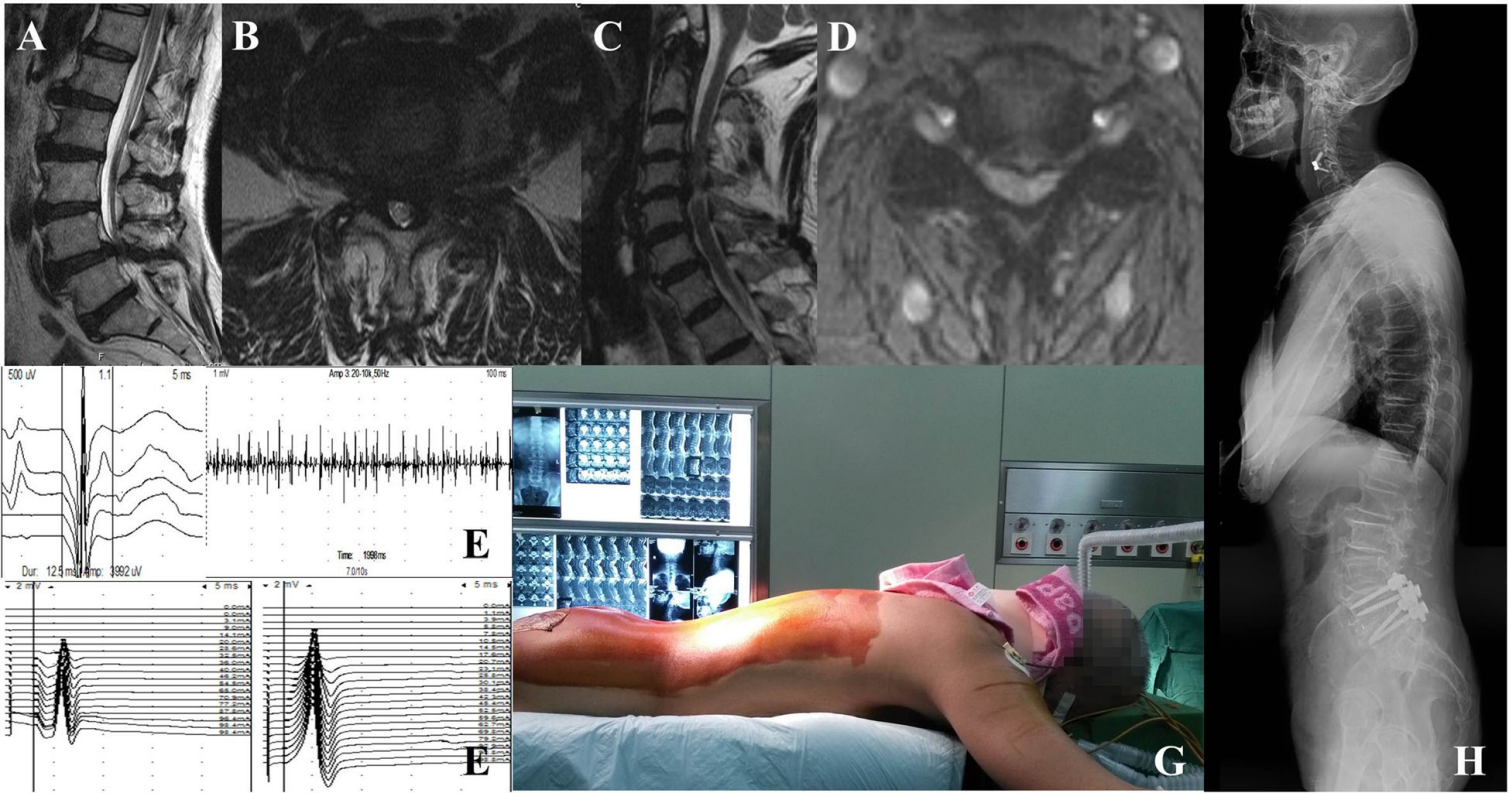
A 68-year-old man had numbness and acratia in the upper extremities and neurogenic claudication. Preoperative magnetic resonance imaging (MRI) showed listhesis at L4 and grade 3 stenosis at L4–5 (**a**, **b**) and disc herniation at C4–5 associated with grade 3 compression (**c**, **d**). The biceps brachii showed motor unit potentials (MUP) with a prolonged duration, increased amplitude, and a simple-mixed phase in the recruitment order (**e**). A bilateral tibial nerve H-reflex was not elicited (**f**). The simultaneous operation was performed using ACDF first and PLIF next, with protection of the neck collar (**g**). Postoperative x-ray of the whole spine showed internal fixators in both the cervical and lumber spine (**h**)

Fourteen patients (10 men and four women) were treated with simultaneous surgery. The ACDF was performed first and then lumbar degenerative disease was treated by TLIF or PLIF in 12 patients (85.71%) or fenestration in two patients (14.29%). A typical case is shown in Fig. 2. All indexes in group CL were significant improved at final follow-up (*p* < 0.001; Table 3).

**Table 3:** Scores in patients underwent cervical and lumbar simultaneous decompression (group CL, *n* = 13)

	Preoperation	Follow-up
Cervical JOA	9.00 ± 1.75	12.29 ± 2.02^1^
NDI	23.14 ± 9.21	9.21 ± 3.85^1^
Lumber JOA	12.21 ± 2.04	19.14 ± 2.57^1^
ODI	27.36 ± 8.25	16.07 ± 5.17^1^

Note: ^1^Compared with preoperation, *P* < 0.05

The comparisons between patients who received both cervical and lumbar surgeries, whether simultaneous or staged, are summarized in Table 4. The gender distribution was similar among the three groups (*p* = 0.639). Group CL was significantly younger (*p* = 0.027), with less coexisting disease (*p* < 0.001), and with the shortest hospitalization time (*p* < 0.001), while there were no differences in these parameters between group C2 and group L2. Group CL showed a trend towards a lower mean ORT and EBL (*p* = 0.138 and *p* = 0.116, respectively). There were significant differences among the three groups in terms of preoperative JOA-C, NDI, JOA-L, and ODI. Before the operation, group L2 had the highest JOA-C and the lowest NDI, while group C2 had the highest JOA-L and the highest ODI. At final follow-up, there were no differences among the three groups in terms of JOA-C, NDI, and ODI. The JOA-L was highest in group C2, while there was no significant difference between group CL and group L2 (*p* = 0.427).

**Table 4:** Comparison of perioperative parameters and follow-up scores in three different groups (group C2, L2, CL)

	Group C2	Group L2	Group CL	*P*
Number	55	26	14	
Age (years)	63.3 ± 7.1	64.6 ± 7.5	58.0 ± 6.7	0.027
Gender				
Male	32	15	10	0.639
Female	23	11	4	
No. of coexisting disease (%)	40 (72.7%)	17 (65.4%)	2 (14.3%)	< 0.001
Operation time (min)	209.64 ± 32.34	224.42 ± 55.40	199.29 ± 35.40	0.138
Blood loss (ml)	489.00 ± 91.21	525.77 ± 63.74	478.57 ± 73.57	0.116
Length of stay (d)	13.44 ± 1.81	14.23 ± 2.14	9.50 ± 1.74	< 0.001
Cervical JOA				
Preoperation	8.55 ± 1.84	10.08 ± 2.10	9.00 ± 1.75	0.005
Follow-up	12.82 ± 2.05	13.50 ± 1.94	12.29 ± 2.02	0.165
NDI				
Preoperation	21.44 ± 8.58	15.50 ± 7.27	23.14 ± 9.21	0.005
Follow-up	12.82 ± 2.05	13.50 ± 1.94	12.29 ± 2.02	0.165
Lumber JOA				
Preoperation	15.35 ± 2.16	11.77 ± 2.16	12.21 ± 2.05	< 0.001
Follow-up	24.51 ± 1.93	19.77 ± 2.23	19.14 ± 2.57	< 0.001
ODI				
Preoperation	30.27 ± 4.94	25.27 ± 8.38	27.36 ± 8.25	0.006
Follow-up	14.36 ± 5.93	13.73 ± 5.83	16.07 ± 5.17	0.475
Complication				0.652
Anemia	2	1	2	
Deep venous thrombosis	2	1	1	
Pneumonia	1	0	0	
Urinary tract infection	2	1	0	

There were no severe intraoperative complications such as incidental durotomy or implant malposition. There were seven patients (12.73%) with postoperative complications in group C2, three patients (11.54%) in group L2, and three patients (21.43%) in group CL (Table 4). There were no significant differences in the rates of complications among the three groups (*p* = 0.652). All these complications were cured and did not affect the final outcome.

## Discussion

### Pathology and surgery in TSS

The true pathogenesis of TSS has not been clearly identified. Congenital stenosis was reported to increase the probability of TSS [13–15]. Lee et al. [14] also examined cervical and lumbar cadaveric specimens of 440 skeletally mature spines and found a prevalence of tandem stenosis of 0.9–5.4%. However, stenosis is most often “acquired” as a result of degenerative disease of the disk, facet joints, and ligamentum flavum, leading to a reduction in spinal canal dimensions [12, 16]. When the neural canal and foramina are compressed by congenital or degenerative spinal stenosis, the resulting symptoms in the cervical and lumber spine appear simultaneously or chronologically. In the present study, all patients were clinico-radiologically diagnosed and the poor preoperative JOA-C, NDI, JOA-L, and ODI scores reflected the associated neurological deficits. Cervical cord compression was treated by ACDF or laminoplasty, while the lumbar symptomatic stenosis or spondylosis was primarily treated by PLIF or TLIF, which can decompress the cauda equine or nerve root and restore lumbar stability. Nine patients received fenestration for unilateral lumbar disc herniation without segment instability. Thus, the primary goal of surgeries in TSS involves decompression, restoring nerve function, and reconstructing spinal stability [12], while the operative technique and selection of the surgical approach shares the same basic principles as for spinal surgery. However, compression in one region may mask the symptoms of compression in other regions. Thus, selection of the optimal surgical strategy remains controversial.

### Choice of staged surgery

Staged surgery was recommended in most studies [2, 3, 12, 17] because of its lower invasiveness and relative security. However, the preferred order of surgical treatment (i.e., cervical first or lumbar first) remains controversial. For example, Dagi et al. [2] performed a retrospective review of 19 patients with a mean follow-up of 22 months and suggested the more symptomatic level should be performed first. Liu et al. [17] suggested that cervical myelopathy had a wider range of effects than lumbar and that the first procedure should be cervical. The proponents of lumbar first suggest that improved lumbar stoop following surgery leads to flexion attitude of the neck, thus creating more space in cervical canal [2].

In the present study, the order of staged surgery was based on the clinical symptoms, signs, and imageological features (Fig. 1). For complicated cases (15 patients in group C2 and seven in group L2), electrophysiological examinations were performed to identify the most clinically relevant level. Cervical decompression was performed first in patients with evidence of myelopathy or primary upper-extremity symptomatology. Grade 3 cervical canal compression (with T2 hyperintensity) was the predominant (75.28%) finding in group C patients, which indicated cord edema with poor prognosis [18]. For patients with intermittent neurogenic claudication or severe leg radiculopathy, lumbar surgery was performed first [19] because intermittent claudication cannot be relieved by cervical decompression [3]. However, if the lumbar stenosis symptoms were more severe than the cervical symptoms, while computed tomography or magnetic resonance imaging revealed more severe cervical canal stenosis than lumbar stenosis, we provide the patients with a detailed outline of the illness and the hazards of probable cervical spondylosis and allow them to choose their preferred first surgery. After the most symptomatic compression was treated, both the JOA-C and NDI in group C and the JOA-L and ODI in group L were significantly improved.

Another advantage of staged surgery is that it allows patients time for recovery of their condition and for making a decision regarding the second surgery, which is dependent on the resolution of symptoms [3, 12]. We suggest that clinicians should pay close attention to the type of symptoms after the first procedure. In the present study, the lower back pain or lumbar radicular symptoms were relieved in 38.20% of patients in group C1, while the second-stage surgery was mainly performed for intermittent claudication of lumbar stenosis in group C2. Only 10.34% of patients in group L1 with neck pain or arm radicular symptoms did not receive a second-stage surgery, while the cervical myelopathic symptoms were exacerbated in group L2. The interval time between staged surgeries was reported as 2 weeks to 1.3 years [20, 21]. In the present study, the mean period was 3.96 ± 1.89 months in group L2, which was significantly shorter than that in group C2 (5.58 ± 1.93 months). The reasons for these differences are as follows. First, the natural history of cervical myelopathy involves a stepwise progression, with any delay in surgery exposing the patient to potentially further loss of function. Second, as the progress of lumbar illness is relatively slow [22], use of a nonsurgical approach, at least initially, is justifiable. Third, the decompression for cervical cord may result in improvement in lumbar symptoms with resolution of pain, spasticity, and sensory deficits of myelopathic origin [20, 23, 24]. Hsieh et al. [25] reported that > 50% of their cohort was treated with cervical decompression in isolation, which mitigated further lumbar surgery. Therefore, we generally addressed the first cervical decompression in staged procedures as it may reduce the requirement for a second lumbar surgery. Nevertheless, if lumbar surgery was performed first, it is important to examine for deterioration due to cervical degenerative myelopathy because the requirement for a second-stage surgery is significantly higher.

### Efficacy in simultaneous surgery

The first one-staged combined surgery for TSS was reported by Dagi et al. [2] in 1987, which involved four patients with short-term follow-up periods. Kikuike et al. [26] reported that 71% of 17 patients were satisfied with single-stage surgery, which was safe for elderly patients. Eskander et al. [27] retrospectively reviewed 21 patients who underwent simultaneous decompression of both the cervical and lumbar spine and 22 patients who underwent staged decompression of the cervical spine followed by the lumbar spine at a later date. In that study, both groups showed improved JOA and ODI, while there were no significant differences among the groups in major or minor complications and for JOA and ODI sat 7-year follow-up. However, an age of > 68 years, an EBL of > 400 mL, and an operative time of >150 min were significantly associated with an increased number of complications. The present study compared simultaneous surgery (group CL) with different sequential staged groups (group C2 and L2) and found that the simultaneous approach resulted in a shorter ORT and less EBL, with no differences in complications. These findings may relate to our strict preoperative assessment of age and the general good condition, disease severity, patient’s high motivation for improvement, and surgeon’s ability. As Eskander et al. [27] reported, age increases the risks of major and minor complications regardless of the surgical algorithm. Furthermore, late outcomes may be influenced by coexisting diseases [10]. Thus, we selected younger patients (58.0 ± 6.7) with fewer comorbidities (14.3%) for the simultaneous operation. There is no standard protocol regarding when motor- and sensory-evoked potentials and electroneuromyography should be added to radiological acquisition in the evaluation of TSS patients [28]. Nevertheless, we recommend performing electrophysiological examinations for every patient receiving simultaneous surgery as it may identify the concomitant cervical and lumbar levels. The single anesthesia in one-staged surgery can help to minimize the length of the hospital stay and associated costs, provide an early return to function, and may be more desirable than exposing comorbid patients to two separate procedures. At final follow-up, all neurological scores were significantly improved in the three groups. The lower JOA-L in group L2 may be associated with the more severe preoperative lumbar illness; however, there were no differences in the JOA-C, NDI, and ODI. As shown in a meta-analysis of 17 studies [12], both staged and simultaneous procedures are efficacious.

Note that surgeons should pay particular attention to the decompression sequence and surgical position in simultaneous surgery. Krishnan et al. [10] reported 53 patients who were operated under general anesthesia in the prone position with a horse-shoe extension for head holding in single-stage surgery. Kikuike et al. [26] reported eight patients who received simultaneous surgery by two surgical teams, while nine patients underwent sequential procedures by a single surgical team. After ACDF, changing to the prone position should be done with caution. Hyperextension positioning during lumbar surgery was reported to cause devastating neurological deficits in patients with cervical stenosis [29]. In the present study, all 14 patients were first positioned supine, followed by leaning on a sponge or a silica gel head holder, which is often used in scoliosis surgery. This can balance the tracheal intubating pronely and preventing of neck hyperflexion or hyperextension, and the skull traction can be avoided. Furthermore, use of neuromonitoring modalities during treatment of severe TSS was recommended [12].

This study had several limitations including its retrospective nature and non-randomized design. Furthermore, the relatively limited number of patients in the simultaneous group did not allow us to investigate the effect of different surgical treatments. Future prospective studies using larger numbers of patients are required to confirm our findings.

## Conclusions

TSS can be effectively managed by either simultaneous or staged decompressions. Staged surgery can be selected based on the main symptomatology and physical exam findings, although treatment of cervical myelopathic symptoms first may be more appropriate. First-stage surgery for cervical stenosis significantly lowers the need for second-stage lumbar surgery. If lumbar stenosis is treated first, cervical symptoms should be monitored closely as they may aggravate over a short period. One-stage simultaneous decompression is safe and effective with the advantage of shorter hospitalization time, with no increase in operative time or bleeding. However, the surgical indications should be strictly controlled. Younger age and fewer comorbidities may be useful factors in choosing simultaneous surgery.

## Data Availability

Data requests are available from the corresponding author.

## Abbreviations

ACDF: Anterior cervical discectomy and fusion
EBL: Estimated blood loss
JOA: Japanese Orthopedic Association
NDI: Neck Disability Index
ODI: Oswestry Disability Index
OPLL: Ossification of the posterior longitudinal ligament
ORT: Operation time
PLIF: posterior lumber interbody fusion
TLIF: Transforaminal lumber interbody fusion
TSS: Tandem spinal stenosis
UMN: Upper motor neuron

## References

[1] Teng P, Papatheodorou C (1964). Combined cervical and lumbar spondylosis. Arch Neurol.

[2] Dagi TF, Tarkington MA, Leech JJ (1987). Tandem lumbar and cervical spinal stenosis. Natural history, prognostic indices, and results after surgical decompression. J Neurosurg.

[3] Epstein NE, Epstein JA, Carras R, Murthy VS, Hyman RA (1984). Coexisting cervical and lumbar spinal stenosis: diagnosis and management. Neurosurgery.

[4] LaBan MM, Green ML (2004). Concurrent (tandem) cervical and lumbar spinal stenosis:a 10-yr review of 54 hospitalized patients. Am J Phys Med Rehabil.

[5] Rahmanian A, Minagar S, Rakei SM (2015). A survey of tandem spinal stenosis in Shiraz, southern Iran. Neurosurg Q.

[6] Hailang S, Huan W, Shaoqian C (2017). Tandem spinal stenosis: current progress and literature review. Orthop J China.

[7] Nagata K, Yoshimura N, Hashizume H, et al. The prevalence of tandem spinal stenosis and its characteristics in a population-based MRI study: the Wakayama spine study. Eur Spine J. 2017;26(10):2529–35. 10.1007/s00586-017-5072-0 28374329

[8] Kang Y, Lee JW, Koh YH, Hur S, Kim SJ, Chai JW, Kang HS (2011). New MRI grading system for the cervical canal stenosis. AJR Am J Roentgenol.

[9] Lee GY, Lee JW, Choi HS, Oh K-J, Kang HS (2011). A new grading system of lumbar central canal stenosis on MRI: an easy and reliable method. Skelet Radiol.

[10] Krishnan A, Dave BR, Kambar AK, Ram H (2014). Coexisting lumbar and cervical stenosis (tandem spinal stenosis): an infrequent presentation. Retrospective analysis of single-stage surgery (53 cases). Eur Spine J.

[11] Park MS, Moon SH, Kim TH, Oh JK, Lyu HD, Lee JH, Riew KD (2015). Asymptomatic stenosis in the cervical and thoracic spines of patients with symptomatic lumbar stenosis. Glob Spine J.

[12] Overley SC, Kim JS, Gogel BA, Merrill RK, Hecht AC (2017). Tandem spinal stenosis: a systematic review. JBJS Rev.

[13] Swanson BT (2012). Tandem spinal stenosis: a case of stenotic cauda equina syndrome following cervicaldecompression and fusionfor spondylotic cervical myelopathy. J Man Manip Ther.

[14] Lee MJ, Garcia R, Cassinelli EH, Furey C, Riew KD (2008). Tandemstenosis: a cadaveric study in osseous morphology. Spine J.

[15] Bajwa NS, Toy JO, Young EY, Ahn NU (2012). Is congenital bony stenosis of the cervical spine associated with lumbar spine stenosis? An anatomical study of 1072 human cadaveric specimens. J Neurosurg Spine.

[16] Kawaguchi Y, Oya T, Abe Y, Kanamori M, Ishihara H, Yasuda T, Nogami S, Hori T, Kimura T (2005). Spinal stenosis due to ossified lumbar lesions. J Neurosurg Spine.

[17] Zhiwei L, Huilin Y, Zhiming Z (2008). The diagnose of cervical and lumbar spinal stenosis and surgical treatment in 22 patients. Suzhou Univ J Med Sci.

[18] Morio Y, Teshima R, Nagashima H, Nawata K, Yamasaki D, Nanjo Y (2001). Correlation between operative outcomes of cervical compression myelopathy and MRI of the spinal cord. Spine.

[19] Aydogan M, Ozturk C, Mirzanli C, Karatoprak O, Tezer M, Hamzaoglu A (2007). Treatment approach in tandem (concurrent) cervical and lumbar spinal stenosis. Acta Orthop Belg.

[20] Felbaum DR, Fayed I, Stewart JJ (2016). Relief of lumbar symptoms after cervical decompression in patients with tandem spinal stenosis presenting with primarily lumbar pain. Cureus.

[21] Yamada T, Yoshii T, Yamamoto N, Hirai T, Inose H, Kato T, Kawabata S, Okawa A (2018). Clinical outcomes of cervical spinal surgery for cervical myelopathic patients with coexisting lumbar spinal canal stenosis (Tandem spinal stenosis): a retrospective analysis of 297 cases. Spine (Phila Pa 1976).

[22] Luo CA, Kaliya-Perumal AK, Lu ML, Chen LH, Chen WJ, Niu CC (2019). Staged surgery for tandem cervical and lumbar spinal stenosis: which should be treated first?. Eur Spine J.

[23] Alvin MD, Alentado VJ, Lubelski D, Benzel EC, Mroz TE (2018). Cervical spine surgery for tandem spinal stenosis: the impact on low back pain. Clin Neurol Neurosurg.

[24] Hai-lang S, Huan W, Shaoqian C (2016). Analyses of clinical outcomes of cervical decompression for patients with tandem spinal stenosis presenting with sciatica-like leg pain. Chin J Bone Joint.

[25] Hsieh CH, Huang TJ, Hsu RW (1998). Tandem spinal stenosis: clinical diagnosis and surgical treatment. Changgeng Yi Xue Za Zhi.

[26] Kikuike K, Miyamoto K, Hosoe H, Shimizu K (2009). One-staged combined cervical and lumbar decompression for patients with tandem spinal stenosis on cervical and lumbar spine: analyses of clinical outcomes with minimum 3 years follow-up. J Spinal Disord Tech.

[27] Eskander MS, Aubin ME, Drew JM (2011). Is there a difference between simultaneous or staged decompressions for combined cervical and lumbar stenosis?. J Spinal Disord Tech.

[28] Jannelli G, Baticam NS, Tizi K, Truffert A, Lascano AM, Tessitore E (2020). Symptomatic tandem spinal stenosis: a clinical, diagnostic, and surgical challenge. Neurosurg Rev.

[29] Chen SH, Hui YL, Yu CM (2005). Paraplegia by acute cervical disc protrusion after lumbar spine surgery. Chang Gung Med J.

